# Characterisation of the *Mentha canadensis* R2R3-MYB transcription factor gene *McMIXTA* and its involvement in peltate glandular trichome development

**DOI:** 10.1186/s12870-022-03614-9

**Published:** 2022-04-28

**Authors:** Xiwu Qi, Zequn Chen, Xu Yu, Li Li, Yang Bai, Hailing Fang, Chengyuan Liang

**Affiliations:** 1 Jiangsu Key Laboratory for the Research and Utilization of Plant Resources, Institute of Botany, Jiangsu Province and Chinese Academy of Sciences, Nanjing, 210014 China; 2 College of Forestry, Nanjing Forestry University, Nanjing, 210037 China

**Keywords:** *Mentha canadensis* L.; Glandular trichome; *McMIXTA*; *McHD-Zip3*

## Abstract

**Background:**

*Mentha canadensis* L. has important economic value for the production of essential oils, which are synthesised, secreted and stored in peltate glandular trichomes. As a typical multicellular secretory trichome, glandular trichomes are important biological factories for the synthesis of some specialised metabolites. However, little is known about the molecular mechanism of glandular trichome development in *M. canadensis*.

**Results:**

In this study, the R2R3-MYB transcription factor gene *McMIXTA* was isolated to investigate its function in glandular trichome development. Bioinformatics analysis indicated that McMIXTA belonged to the subgroup 9 R2R3-MYB, with a R2R3 DNA-binding domain and conserved subgroup 9 motifs. A subcellular localisation assay indicated that McMIXTA was localised in the nucleus. Transactivation analysis indicated that McMIXTA was a positive regulator, with transactivation regions located between positions N253 and N307. Yeast two-hybrid and bimolecular fluorescence complementation assays showed that McMIXTA formed a complex with McHD-Zip3, a trichome development-related HD-ZIP IV transcription factor. Overexpression of McMIXTA in *Mentha* × *piperita* L. caused an increase in peltate glandular trichomes density of approximately 25% on the leaf abaxial surface.

**Conclusions:**

Our results demonstrated that the subgroup 9 R2R3-MYB transcription factor McMIXTA has a positive effect on regulating peltate glandular trichome development and the MIXTA/HD-ZIP IV complexes might be conserved regulators for glandular trichome initiation. These results provide useful information for revealing the regulatory mechanism of multicellular glandular trichome development.

## Background

Trichomes are appendages derived from cells of the aerial epidermis, and are found in numerous plant species worldwide [1]. Trichomes vary in size, morphology, cellular composition, and function, and can be categorised into unicellular and multicellular or secretory and non-secretory types according to their cell number and secretory ability [2, 3]. For instance, *Arabidopsis thaliana* trichomes are unicellular and non-secretory [4]. Secretory or glandular trichomes synthesise or secrete large amounts of specialised metabolites that enhance plant fitness and may also be beneficial for humans [5, 6]. For example, glandular trichomes of *Artemisia annua* secrete the sesquiterpene metabolite artemisinin, which is an effective anti-malarial drug [7].

The amounts of metabolites secreted by glandular trichomes are generally correlated to their density [7, 8]. Increased glandular trichome density is considered to be an effective strategy to increase the accumulation of specialised metabolites [7]. Therefore, the genetic networks of glandular trichome development provide an important basis for genetic engineering of glandular trichome. In *A. thaliana*, more than 70 genes regulating trichome development have been identified and characterised, revealing a complex regulatory network for trichome [9–14]. In contrast to the unicellular trichomes of *Arabidopsis*, the regulatory networks of multicellular glandular trichome development in other species remain poorly understood [15]. To date, only a few genes regulating multicellular glandular trichome development have been identified, which mainly encode some transcription factors, including MYB, HD-ZIP, C2H2, bHLH and so on [15]. The *MIXTA* gene, which was first characterised in *Antirrhinum majus*, encodes one of the most important regulators of glandular trichomes and promotes trichome initiation [16–18]. Homologous genes have also been identified in *A. annua* and *Solanum lycopersicum*, which have been shown to have similar functions in promoting glandular trichome initiation [19, 20].

In addition to *MIXTA* genes, several HD-ZIP IV transcription factors have also been shown to participate in regulating glandular trichome development [15]. For example, AaHD1 and AaHD8 in *A. annua* can promote the development of glandular trichome [21, 22]. In *S. lycopersicum*, SlWOOLLY and SlCD2 have been shown to participate in regulation of glandular trichome initiation [23–25]. Further, there is evidence that the glandular trichome-related MIXTA and HD-ZIP transcription factors may function through interaction. In *A. annua*, AaMIXTA1 and AaHD8 show similar functions in promoting glandular trichome initiation, and they interact to form complexes. The interaction of AaMIXTA1 and AaHD8 produces a much greater effect than their additive effects [22]. In *S. lycopersicum*, SlMIXTA-like and SlCD2 may also interact to form a co-regulation network [15].

*Mentha canadensis* L. (Lamiaceae) can produce a large amount of aromatic essential oils, which are widely used in food additives, cosmetics and medical industries [26]. The main components of essential oils are monoterpenoids, which are synthesised, secreted, and stored in glandular trichomes [27, 28]. The biosynthetic pathway of monoterpenoids in essential oils has been extensively studied in the genus *Mentha* [29–31]. However, the genetic mechanism of the involvement of glandular trichomes in essential oil biosynthesis has rarely been explored. The surface of *M. canadensis* bears three types of trichomes: non-glandular, capitate glandular, and peltate glandular trichomes; among these, peltate glandular trichomes are the centre of essential oil biosynthesis [32]. In our previous study, we demonstrated that McHD-Zip3, a HD-Zip IV transcription factor of *M. canadensis*, participated in promoting glandular trichome development [33]. However, the regulation mechanism of glandular trichome development in *M. canadensis* requires further study.

Therefore, in this study, we cloned the *MIXTA* homologue gene *McMIXTA* from *M. canadensis* and characterised its regulatory role in glandular trichome development. We further examined whether McMIXTA interacted with the HD-ZIP IV transcription factor McHD-Zip3 to form a complex. The results of this study will contribute to elucidating the molecular mechanism of multicellular glandular trichome development in *M. canadensis*.

## Results

### McMIXTA is a subgroup 9 R2R3-MYB transcription factor in *M. canadensis*

**Fig. 1:**
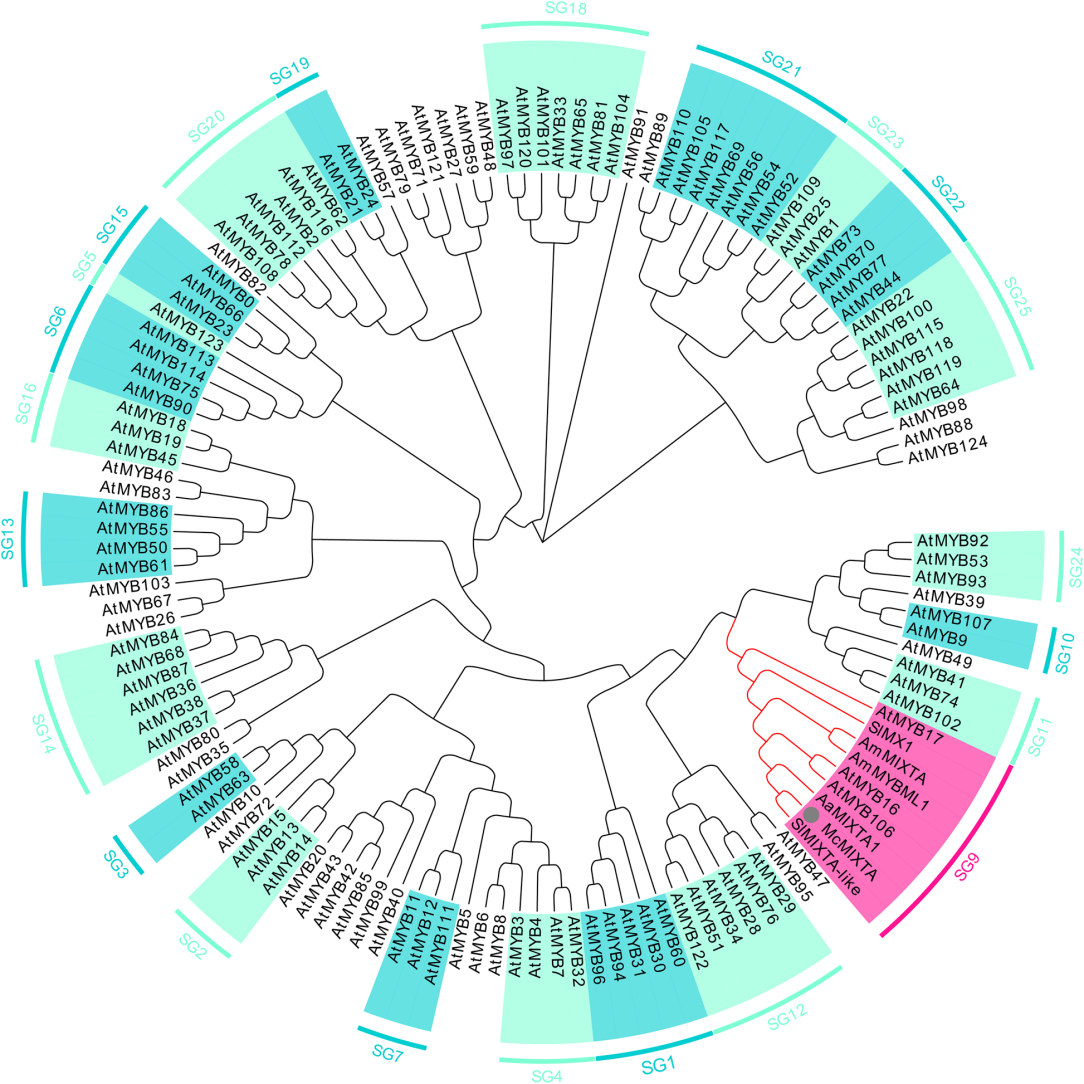
Phylogenetic analysis of McMIXTA, Arabidopsis R2R3-MYBs and representative subgroup 9 MYBs in other plants. ﻿The Arabidopsis R2R3-MYB sequences were accessed from TAIR (https://www.arabidopsis.org/ ). The representative subgroup 9 MYBs are AmMIXTA and AmMYBML1 from *A. majus*, AaMIXTA1 from *A. annua*, and SlMX and SlMIXTA-like from *S. lycopersicum*

**Fig. 2:**
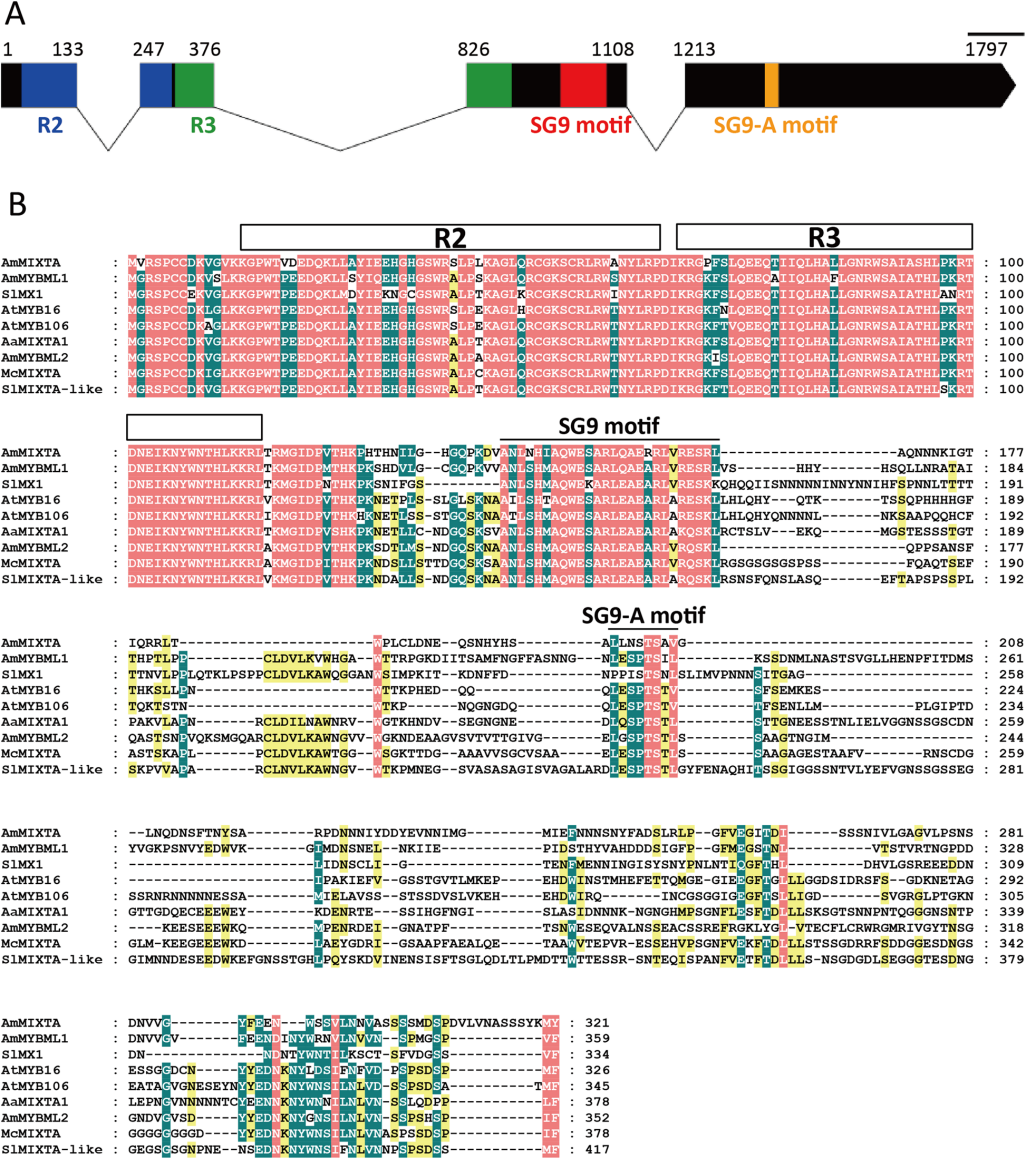
The exon–intron structure and multi-sequence alignment of McMIXTA. **A** The exon–intron structure of McMIXTA. Exons and introns are represented by rectangles and broken lines respectively. The blue, green, red and orange rectangles represent R2, R3, SG9 motif and SG9-A motif, respectively. **B** Multi-sequence alignment of McMIXTA and other subgroup 9 R2R3-MYBs. Proteins used for alignment are AtMYB16 (AT5G15310) and AtMYB106 (AT3G01140) from *A*. *thaliana*, AmMIXTA (CAA55725.1), AmMYBML1 (CAB43399.1), and AmMYBML2 (AAV70655.1) from *A*. *majus*, AaMIXTA1 (ALD84252.1) from *A*. *annua*, and SlMX (Solyc01g010910) and SlMIXTA-like (Solyc02g088190) from *S*. *lycopersicum*. The *A*. *thaliana* sequences are downloaded from TAIR (https://www.arabidopsis.org/) and the *S*. *lycopersicum* sequences are downloaded from Sol Genomics Nerwork (https://solgenomics.net/)

In this study, we isolated the homologous gene of *MIXTA* in *M. canadensis*, which we named *McMIXTA* (GenBank accession number: OL624641). Phylogenetic analysis showed that McMIXTA was clustered into one clade with other subgroup 9 MYB members, most of which have been characterised as regulators associated with epidermal cell differentiation (Fig. 1). A comparison of the CDS and corresponding genome sequence showed that *McMIXTA* contained four exons and three introns (Fig. 2A). The CDS of *McMIXTA* was 1137 bp in length, which encoded a 337 amino acid protein with a theoretical molecular weight of 40.7 kDa and a theoretical isoelectric point of 5.97. Multi-sequence alignment showed that McMIXTA shared a conserved R2R3 repeat region and subgroup 9-specific motifs SG9 and SG9-A with other subgroup 9 R2R3-MYBs (Fig. 2B). These results suggest that McMIXTA is a subgroup 9 R2R3-MYB transcription factor in *M. canadensis*.

### McMIXTA is a nuclear-localised protein

**Fig. 3:**
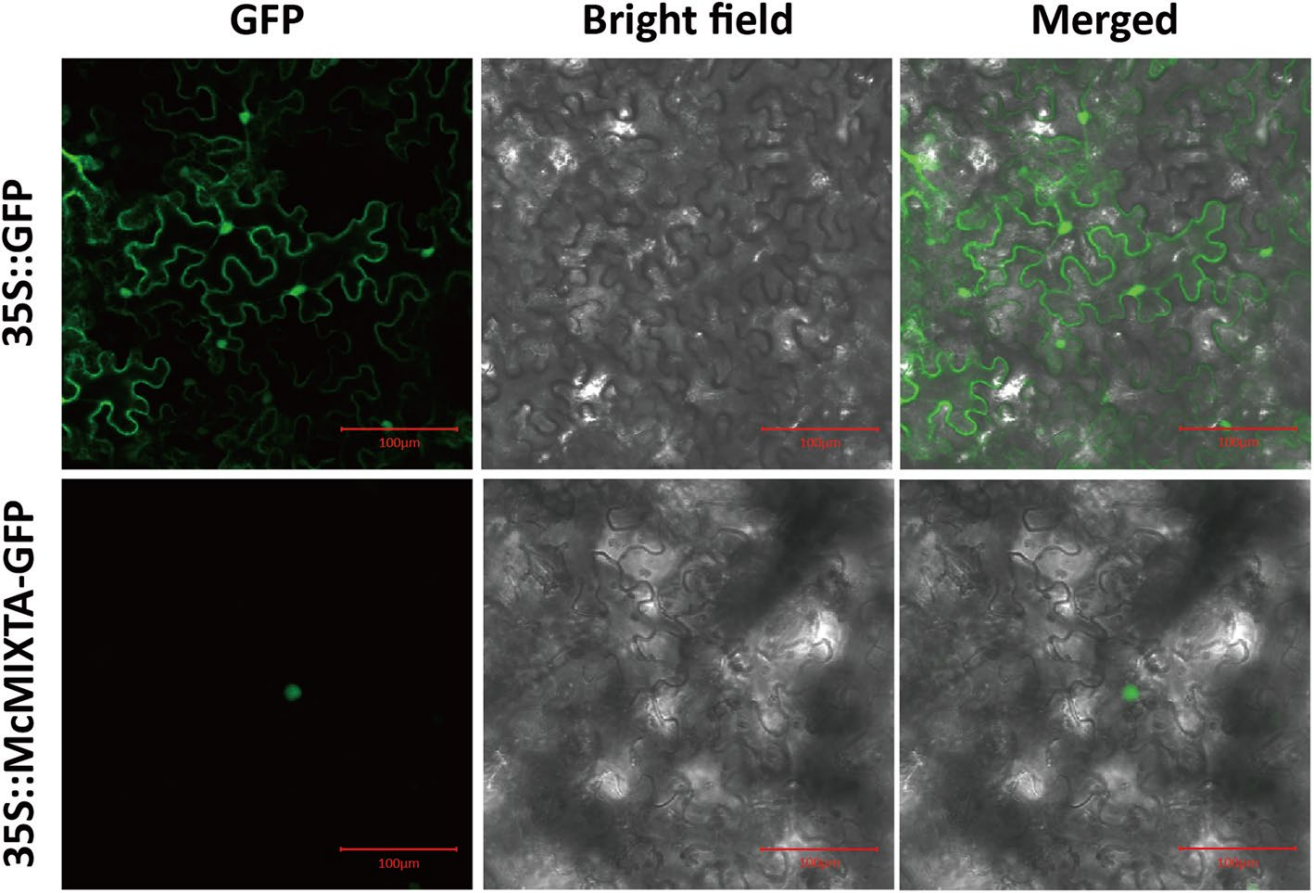
Subcellular localization of the McMIXTA protein in tobacco epidermal cells

Transcription factors mainly perform regulatory functions in the nucleus. In this study, *Agrobacterium*-mediated transient transformation in *Nicotiana benthamiana* epidermal cells was conducted to analyse the subcellular localisation of McMIXTA. Using fusion-expressed GFP as a marker, we found that the McMIXTA-GFP fusion protein was present only in the nucleus, whereas GFP was present in the whole cell (Fig. 3). These results suggest that McMIXTA is localised in the nucleus.

### The McMIXTA N253–307 region is sufficient for transactivation

**Fig. 4:**
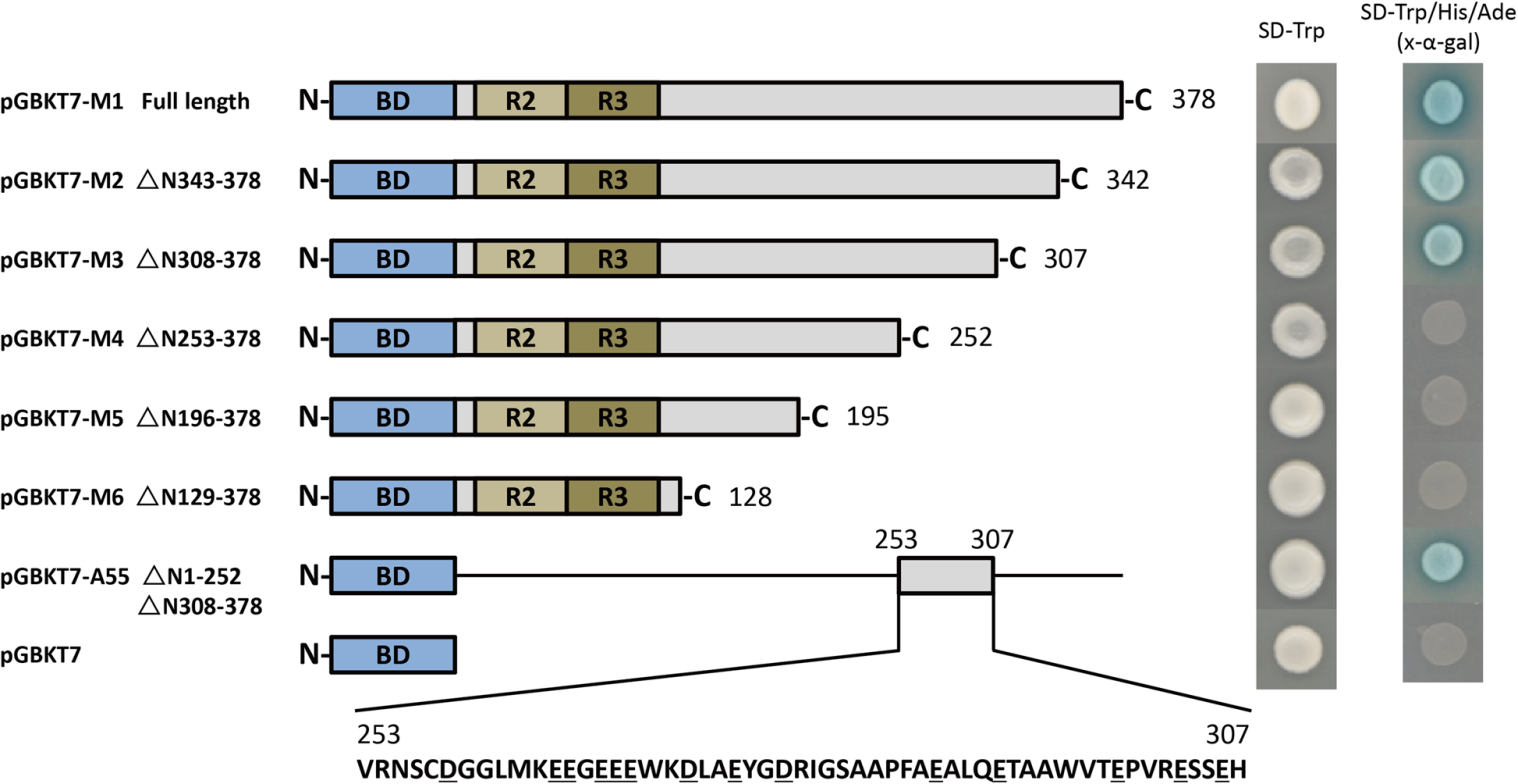
Deletion analysis for screening the transcriptional activation regions of McMIXTA. Different parts of McMIXTA were fused with the GAL4 DNA-binding domain and transformed into yeast strain AH109 containing the *HIS3* and *LacZ* reporter genes (The underlined bases are acidic amino acids)

Yeast cells were used to identify the transactivation region of McMIXTA. The full-length CDS and a series of C-terminal truncated CDSs of McMIXTA were fused separately to the GAL4 DNA BD in pGBKT7 vector and transformed into yeast strain AH109. Screening medium and a LacZ activity assay were used to detect the transactivation activity of the recombinant plasmids. The results showed that three forms of McMIXTA with amino acid length > 307 (M1 Full length, M2 ΔN343–378, and M3 ΔN308–378), had transcriptional activation activity, whereas the other three forms with amino acid length < 252 (M4 ΔN253–278, M5 ΔN196–378, and M6 ΔN129–378) did not (Fig. 4), suggesting that the transcriptional activation regions of McMIXTA may be located between N253 and N307. To confirm whether the region between positions N253 and N307 of McMIXTA was sufficient for transactivation, these regions were cloned into the pGBKT7 vector and assayed using yeast cells. The results indicate that region N253–307 of McMIXTA is sufficient for transactivation (Fig. 4).

### Overexpression of *McMIXTA* promotes peltate glandular trichome initiation in *M. piperita*

**Fig. 5:**
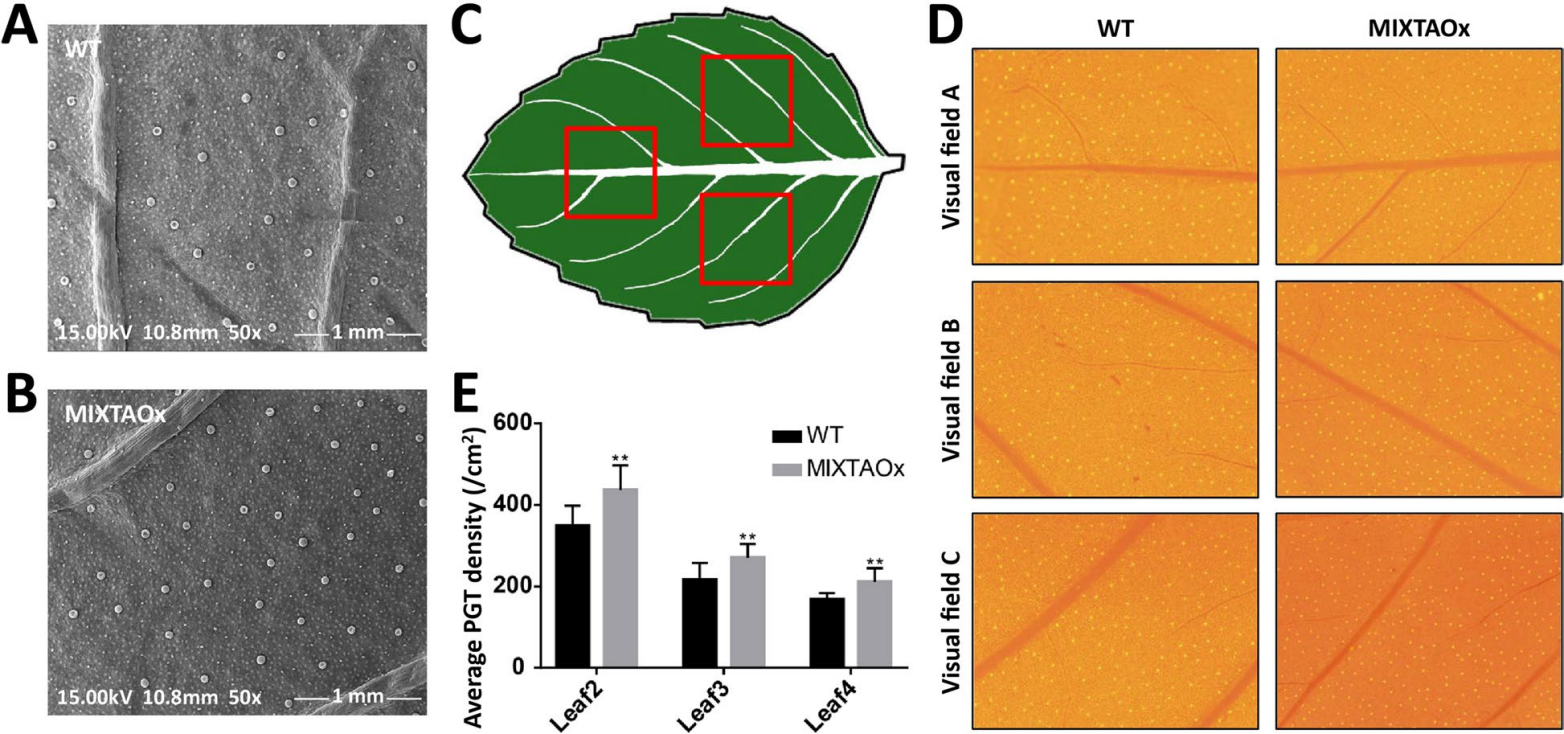
Overexpression of *McMIXTA* promotes the initation of peltate glandular trichomes in *M. piperita* leaves. **A** and **B** SEM observation of peltate glandular trichomes on abaxial side of WT and *McMIXTA* overexpression (MIXTAOx) *M. piperita* leaves, respectively. **C** Three relatively fixed visual fields per leaf were obtained to represent the average peltate glandular trichome density. **D** The peltate glandular trichomes on the abaxial side of leaves derived from WT and *McMIXTA* overexpression *M. piperita* leaves. **E** Density of peltate glandular trichomes on abaxial side of leaves derived from WT and *McMIXTA* overexpression plants

To determine whether *McMIXTA* has an effect on peltate glandular trichome initiation in mint plants, the 35S::McMIXTA overexpression vector was introduced into *M. piperita*, a *Mentha* plant with similar glandular trichome structure to *M. canadensis*. After screening transgenic plants by antibiotic selection and β-glucuronidase staining, scanning electron microscopy (SEM) was conducted to visualise the peltate glandular trichomes on abaxial side of WT and *McMIXTA* overexpression *M. piperita* leaves. The results showed that there was no difference in the morphology and size of peltate glandular trichomes in *McMIXTA* overexpressed plants compared with WT plants, but the density of peltate glandular trichome increased (Fig. 5A, B). Due to the small field of SEM, we further used stereo fluorescence microscopy to count the number of peltate glandular trichomes on leaves, in order to obtain more accurate data. Because peltate glandular trichomes are not evenly distributed on leaves, we obtained images of three relatively fixed visual fields per leaf to represent the average peltate glandular trichome density of the leaf (Fig. 5C). Peltate glandular trichome count results showed that the density of peltate glandular trichome decreased gradually with the growth of leaves (Fig. 5E). Comparing transgenic plants with wild-type plants showed that the second, third, and fourth leaves of *McMIXTA* overexpressed transgenic plants had significantly higher peltate glandular trichome density than those of the WT and the increase in peltate glandular trichome density was about 25% (Fig. 5D, E). These results suggest that overexpression of *McMIXTA* promotes the initiation of peltate glandular trichomes in *M. piperita*.

### McMIXTA interacted with the glandular trichome development-related transcription factor McHD-Zip3

**Fig. 6:**
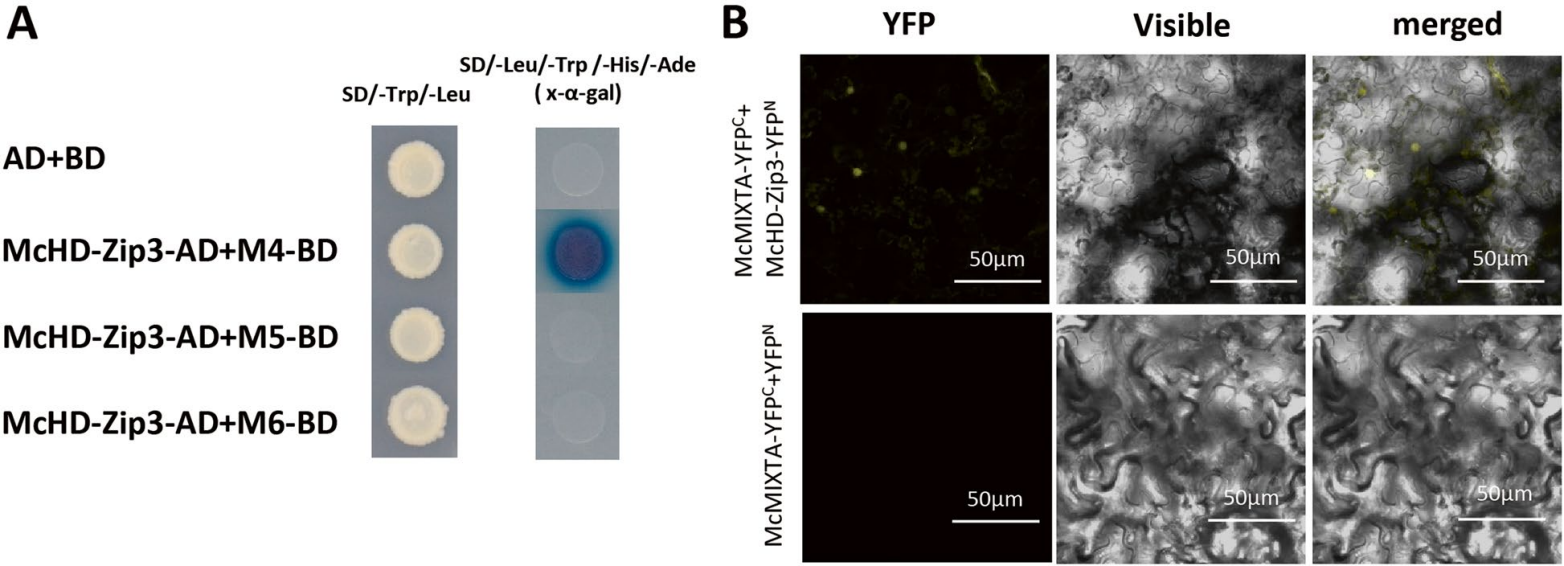
McMIXTA interacts with McHD-Zip3. **A** Yeast two-hybrid assay of McHD-Zip3 and McMIXTA with different truncated lengths. **B** BiFC analysis of McHD-Zip3 and McMIXTA. McMIXTA-YFP^C^ vector and empty YFP^N^ vector were used as negative controls

Studies have shown that MIXTA interacts with HD-ZIP IV transcription factors to form complexes that regulate epidermal cell development [15, 22]. In our previous study, we identified the HD-ZIP IV transcription factor McHD-Zip3 in *M. canadensis*, which promoted glandular trichome development [33]. Therefore, we attempted to detect an interaction between McMIXTA and McHD-Zip3 in this study. Due to strong transcriptional self-activation of McMIXTA, we performed a deletion analysis; we found that the AD was localised in residues 252–307 at the C-terminus. Consequently, M4, M5, and M6 were fused to the yeast GAL4 BD and McHD-Zip3 was fused to the yeast GAL4 AD. Results of a Y2H assay showed that M4 interacted with McHD-Zip3, but M5 and M6 did not (Fig. 6A), indicating that McMIXTA interacted with McHD-Zip3 in vitro and that N195–252 of McMIXTA may be the key region binding to McHD-Zip3. BiFC experiments also demonstrated that McMIXTA and McHD-Zip3 interacted in vivo (Fig. 6B).

## Discussion

In the present study, we identified the R2R3-MYB transcription factor gene *McMIXTA* in *M. canadensis*. Phylogenetic analysis and conserved motif analysis indicated that *McMIXTA* belongs to subgroup 9 R2R3-MYB. A subcellular localisation assay showed that McMIXTA was localised in the nucleus. Transactivation analysis indicated that McMIXTA was a positive regulator, the transactivation regions of which were located between positions N253 and N307. Overexpression of McMIXTA in *M. piperita* increased peltate glandular trichome density on the leaf abaxial surface. We also demonstrated that McMIXTA interacted with McHD-Zip3 to form a complex and N195–252 of McMIXTA may be the key region for binding.

A transcription factor generally consists of a DNA BD and a transcription regulatory domain that harbours an AD or repression domain. Studies have shown that R2R3-MYB transcription factors share a conserved N-terminal DNA BD and a transcription regulatory domain at the C-terminal [34]. Nevertheless, few experiments have been performed to elucidate the transactivation domain in R2R3-MYB transcription factors. The transactivation region of R2R3-MYB transcription factors is located at the rearmost C-terminal end of the protein. For example, the transactivation region of *Zea mays* ZmC1 is located in amino acids 234–273 (of 273) [35] and AtMYB2 is located in amino acids 221–274 (of 274) [36]. However, the transactivation region of AtMYB12 is not located at the rearmost C-terminal end but rather near the C-terminus (amino acids 273–325, of 371) [37]. Our results showed that the transactivation region of McMIXTA is located in amino acids 253–307 (of 378), which is farther from the C-terminal end than those of previously reported R2R3-MYB transcription factors. Although the location is different, the transactivation region of McMIXTA is rich in acidic amino acids, which is consistent with other R2R3-MYB transcription factors [35–37].

R2R3-MYB transcription factors represent a large gene family that can be divided into more than 22 subgroups according to the conservation motifs of the C-terminal [34, 38]. Several subgroup 9 R2R3-MYB transcription factors have been shown to regulate epidermal cell differentiation. In *Arabidopsis*, AtMYB16 and AtMYB106 (subgroup 9) have been shown to function in controlling epidermal regulation and trichome branch formation [39, 40]. AaMIXTA1 is a subgroup 9 R2R3-MYB that positively regulates glandular trichome initiation in *A. annua* [19]. In *S. lycopersicum*, two subgroup 9 R2R3-MYBs, SlMX1 and SlMIXTA-like, also participate in regulating trichome development [20, 41]. GhMYB25 and GhMYB25-like (Subgroup 9) regulate the initiation and timing of initial fibre expansion in cotton [42, 43]. In this study, McMIXTA overexpression resulted in higher glandular trichomes density in *M. piperita*, indicating that McMIXTA functions similarly in the regulation of trichome development to other subgroup 9 homologues. However, different types of trichomes have different transcriptional regulatory patterns. For example, the *MIXTA* gene in *A. majus* promotes the development of multicellular glandular trichomes in *N. benthamiana*, but has no effect on unicellular trichomes in *Arabidopsis* [44]. In contrast, *Arabidopsis* AtMYB16 regulates the development of unicellular trichomes but has no effect on multicellular trichomes [39]. These results suggest that although the functions of subgroup 9 members are conserved to some extent, differences remain in the regulation of different types of glandular trichomes.

Studies have shown that MIXTA interacts with HD-ZIP IV transcription factors to form complexes that regulate epidermal cell development. In cotton, the MIXTA-like transcription factor GhMYB25 and HD-ZIP IV transcription factor GhHD1 interact with each other, and both have a positive effect on regulating trichome initiation in fibres [45]. In *A. annua*, AaMXITA1 and AaHD8 have similar functions in the positive regulation of glandular trichome initiation, and interact with each other to form complexes. In *S. lycopersicum*, a co-regulation network between SlMIXTA-like and SlCD2 may also be formed through interaction [15]. In the present study, we also found that McMIXTA interacted with the HD-ZIP IV transcription factor McHD-Zip3 and both of them functions in regulating glandular trichome development [33]. However, the effect of the interaction between MIXTA and HD-ZIP IV on glandular trichome development is rarely reported. In *A. annua*, the activation of AaHD8 to target promoters was significantly enhanced by AaMIXTA1, similarly, the activation of AaMIXTA1 to target promoters was also enhanced by AaHD8, and the enhancement was more than addition of their individual promotion. However, whether this enhancement on target genes can promote more glandular trichomes remains unclear [22]. The interaction between MIXTA and HD-Zip IV transcription factors on glandular trichome development still needs further study. In this study, we demonstrated that N195–252 of McMIXTA may be the key region binding to McHD-Zip3, which is consistent with the AaMIXTA1 in *A. annua*. The C-terminal region of AaMIXTA1 is required for its interaction with AaHD8, whereas the R2R3 domain is not needed. Together, it is possible that MIXTA/HD-ZIP IV complexes are conserved regulators of epidermal cell development in plants and play major roles in trichome initiation.

## Materials and methods

### Plant materials

The *M. canadensis* and *Mentha* × *piperita* L. plants used in this study were maintained at the Germplasm Nursery at the Institute of Botany, Jiangsu Province and the Chinese Academy of Sciences (Nanjing Botanical Garden Mem. Sun Yat-Sen), Nanjing, Jiangsu Province. Leaves of *M. canadensis* were collected and stored at − 80 °C until further use. *N. benthamiana* plants were cultured in an artificial climate chamber (Jiangnan, Ningbo, China) at 25 °C under a 14-h light/10-h dark cycle (120 μmol/m^2^/s).

### RNA isolation and cDNA synthesis

Total RNA of *M. canadensis* and *M. piperita* samples was extracted using the FastPure Plant Total RNA Isolation Kit (Vazyme, Nanjing, China) according to the manufacturer’s instructions. RNA quality and concentration were measured using a NanoDrop 2000 spectrophotometer (Thermo Scientific, Waltham, MA, USA). Next, 1 μg of RNA was used to synthesise first-strand cDNA using the HiScript II 1st Strand cDNA Synthesis Kit (Vazyme).

### Isolation and characterisation of McMIXTA

The candidate gene *McMIXTA* was isolated by polymerase chain reaction (PCR) amplification using *M. canadensis* cDNA as template and primers listed in Table 1. The PCR products were separated by gel electrophoresis and the target fragments were purified and cloned using the 5 min TA/Blunt-Zero Cloning Kit (Vazyme). Positive clones were screened and sequenced by Sangon Biotech (Shanghai) Co., Ltd. for confirmation. The deduced amino acid sequence and corresponding theoretical molecular weights and isoelectric points of *McMIXTA* were predicted in ExPASy (https://www.expasy.org/) [46]. Muscle3.6 was used to conduct the multiple sequence alignment [47]. The neighbour-joining phylogenetic tree was constructed using MEGA4 software with bootstrap testing of 1,000 replications [48]. The bootstrap consensus tree was exported in the Newick file format and modified using the EvolView online tool (https://www.evolgenius.info//evolview/#login) [49].

**Table 1:** Primer sequences used in this study

Appliacation	Primer name	Primer sequence
Gene cloning	*McMIXTA-F*	5'-GTTTTGGTATTGCATCTCGTG-3'
	*McMIXTA-R*	5'-AGCTGATACATTTGAATTTTTGG-3'
Subcellular Localisation Assay	*McMIXTA-SL-F*	5’-TGGAGAGGACAG*GGTACC*ATGGGCAGGTCTCCGTGTT-3’
	*McMIXTA-SL-R*	5’-CTTGCTCACCAT*GGTACC*AAATATCGGCGAATCAGATG-3’
Transactivation Analysis	*McMIXTA-378-F*	5’-GAGGAGGACCT*GCATAT*GATGGGCAGGTCTCCGTGTT-3’
	*McMIXTA-378-R*	5’-ACGGATCCCCGG*GAATTC*TCAAAATATCGGCGAATCAG-3’
	*McMIXTA-342-F*	5’-GGAATTC*CATATG*ATGGGCAGGTCTCCGTGTT-3’
	*McMIXTA-342-R*	5’-CG*GGATCC*GCTGCCGTTATCCGATTC-3’
	*McMIXTA-307-R*	5’-CG*GGATCC*GTGCTCCGAGCTCTCCCG-3’
	*McMIXTA-252-R*	5’-CG*GGATCC*GAACGCCGCCGTGCTCTC-3’
	*McMIXTA-195-R*	5’-CG*GGATCC*CTTCGAAGTGGAGGCGAATTC-3’
	*McMIXTA-128-R*	5’-CG*GGATCC*GGGTTTATGGGTAATCGGGTC-3’
Y2H assay	*McHDZIP3-AD-F*	5’-ATGGAGGCCAGTGAATTCATGGAGAATCTTGGAGAGATGG-3’
	*McHDZIP3-AD-R*	5’-CCCACCCGGGTGGAATTCTCAGTTGCATTGAAGCCCAG-3’
BiFc assay	*McMIXTA-NY-F*	5’-GGAATTC*CATATGATG*GGCAGGTCTCCGTGTT-3’
	*McMIXTA-NY-R*	5’-CG*GGATCC*GAACGCCGCCGTGCTCTC-3’
	*McHDZIP3-CY-F*	5’-ATCCTCTAGA*GTCGAC*ATGGAGAATCTTGGAGAGATGG-3’
	*McHDZIP3-CY-R*	5’-TGCCTGCAG*GTCGAC*GTTGCATTGAAGCCCAGCT-3’
*M. piperita* transformation	*McHDZIP3-OE-F*	5’- GGGTACCCGGGGATCCATGGGCAGGTCTCCGTGTT-3’
	*McHDZIP3-OE-R*	5’-ATTCCTGCAGGTCGACTCAAAATATCGGCGAATCAG-3’

The underline indicates the homologous arm and the italic indicates the restriction site

### Subcellular localisation assay of McMIXTA

For subcellular localisation assay, coding sequences (CDSs) of *McMIXTA* were amplified and cloned into the p35SGK- GFP vector using the ClonExpress II One Step Cloning Kit (Vazyme). Primer sequences were listed in Table 1. The recombinant plasmid was transformed into *Agrobacterium tumefaciens* strain EHA105, and the p35SGK-GFP vector without *McMIXTA* was transformed into *A. tumefaciens* strain EHA105 as a control. Transient transformation was conducted by infiltrating *N. benthamiana* leaves with *A. tumefaciens* strain EHA105 expressing McMIXTA-GFP or GFP only. After 48 h, a laser confocal microscope (Zeiss, Oberkochen, Germany) was used to observe the GFP signals.

### Transactivation activity analysis of McMIXTA

The transactivation activity of McMIXTA was analysed in yeast cells as previously described [50]. To determine the transcriptional activation region of McMIXTA, the full-length CDS of *McMIXTA* and a series of C-terminal truncated CDSs of *McMIXTA* were cloned into the pGBKT7 vector (Clontech). The recombinant plasmids were transformed into yeast strain AH109 and cultured on SD/-Trp medium. Then, the yeast cells were further screened on SD/-Trp or SD/-Trp/-His/-Ade plus 3-AT medium. LacZ activity was assayed by adding X-α-gal to the medium. After the transactivation regions were screened, the identified regions were cloned into the pGBKT7 vector; the yeast assay was also used to detect independent transactivation activity. Primers used for vector construction are listed in Table 1.

### Yeast two-hybrid (Y2H) assay

For the Y2H assay, the CDS of *McHD-Zip3* was amplified and cloned into pGADT7 to generate the GAL4 activation domain (AD) vector using primers listed in Table 1. pGBKT7 inserted with full-length or truncated CDSs of *McMIXTA* was used as the GAL4 BD vector. The Y2H assay was conducted by co-transformation of the AD and BD vectors to yeast strain AH109 and cultured on SD/-Trp/-Leu medium. Then, the yeast cells were screened on SD/-Trp/-Leu or SD/-Ade/-His/-Leu/-Trp medium. X-α-gal was added to the medium to detect the activity of the reporter gene *MEL1*.

### Bimolecular fluorescence complementation (BiFC) assay

The pXY103 and pXY105 vectors containing N-terminal and C-terminal CDSs of yellow fluorescent protein (YFP) were used for the BiFC assay. CDSs of *McHD-Zip3* and *McMIXTA* were fused to the N-terminal and C-terminal of YFP, respectively, using primers listed in Table 1. The recombinant vectors were transformed into *A. tumefaciens* strain EHA105. After *A. tumefaciens* culturing, different combinations of nYFP and cYFP bacterial solutions were co-infiltrated into *N. benthamiana* leaves. After 48 h, the fluorescence signals were observed using a laser confocal microscope (Zeiss).

### Genetic transformation of *M. piperita*

CDSs of *McMIXTA* were cloned into the plant binary expression vector p35SGK using the ClonExpress II One Step Cloning Kit (Vazyme) using primers listed in Table 1. The expression of the inserted gene was controlled by the *CaMV 35S* promoter. The recombinant plasmid was transformed into *A. tumefaciens* EHA105 competent cells. Transformation of *M. piperita* was conducted as previously described [51], with some modification. Briefly, sterile stems were used as explants and pre-cultured on pre-culture medium for 2 days. Then, the explants were submerged in *A. tumefaciens* containing the recombinant plasmid p35SGK-McMIXTA and incubated for 20 min with gentle shaking. The explants were dried with sterilised blotting paper and cultured in co-cultivation medium for 2 days in the dark. Then, the explants were transferred to screening medium containing 50 mg/L kanamycin and 25 mg/L rifampicin and cultured at 25 °C under a 16-h light/8-h dark cycle. After 6 weeks, resistant plants differentiated from explants on the screening medium were transferred to rooting medium. Well-rooted plants were then transferred to vegetative soil for culture.

### Scanning electron microscopy (SEM)

The peltate glandular trichomes of transgenic and wild-type (WT) *M. piperita* were observed using scanning electron microscopy. For sample preparation, the second leaves were fixed overnight in 2.5% glutaraldehyde prepared in 0.1 M potassium phosphate buffer. Then the samples were washed with distilled water and dehydrated in an ethanol and acetone series. After dehydration, the samples were critical point dried and fixed on spherical metal stubs. The abaxial surface of leaves was coated with a thin layer of gold and observed using FEI Quanta 200 scanning electron microscope (FEI, Hillsboro, USA).

### Peltate glandular trichome density counts

The second, third, and fourth leaves of transgenic and WT *M. piperita* were used to determine peltate glandular trichome density. Peltate glandular trichomes on the leaf abaxial surface were observed under a stereo fluorescence microscope (Olympus, Tokyo, Japan) with a × 5 objective at an excitation wavelength of 480 nm. The peltate glandular trichome number was determined using the ImageJ software. Because peltate glandular trichomes are not evenly distributed on leaves, we obtained images for three relatively fixed visual fields per leaf to represent the average peltate glandular trichome density of the leaf. The leaves of six plants were measured for both transgenic and WT lines.

We declare that all methods were carried out in accordance with relevant guidelines and regulations

## Data Availability

The datasets generated and analysed during the current study are available in the NCBI GenBank with accession numbers of OL624641 (McMIXTA), CAA55725.1 (AmMIXTA), CAB43399.1 (AmMYBML1), AAV70655.1 (AmMYBML2) and ALD84252.1 (AaMIXTA1). The MYB transcription factor family of *A. thaliana* are available in TAIR (https://www.arabidopsis.org/browse/genefamily/myb-agris.jsp). The SlMX and SlMIXTA-like sequences of *S. lycopersicum* are available in Sol Genomics Nerwork (https://solgenomics.net/) with accession numbers of Solyc01g010910 and Solyc02g088190, respectively.
